# A Systematic Review of Pharmacists Using Motivational Interviewing and Patient Outcomes

**DOI:** 10.3390/pharmacy14060136

**Published:** 2026-09-17

**Authors:** Blair Woodard, Angelia LaPresta, Baylee Eastes, Stephanie M. Tubb, Aleda M. H. Chen

**Affiliations:** 1 School of Pharmacy, Cedarville University, Cedarville, OH 45314, USA; blairwoodard@cedarville.edu (B.W.); alapresta@cedarville.edu (A.L.); bayleeeastes@cedarville.edu (B.E.); smtubb@cedarville.edu (S.M.T.); 2 College of Pharmacy, University of Arkansas for Medical Sciences, Little Rock, AR 72205, USA

**Keywords:** motivational interviewing, pharmacists, students, pharmacy, medication adherence, patient outcome assessment, chronic disease, counseling, systematic review

## Abstract

Background: Patients often must change their behaviors to prevent and mitigate the impact of chronic disease, yet deciding to begin and adhere to these changes can be challenging. Motivational interviewing (MI), a patient-centered communication approach, has been utilized by pharmacists to support patients in making these changes. Thus, this systematic review aimed to describe the outcomes of MI interventions by pharmacists and student pharmacists. Methods: Using PRISMA methodology and registered in PROSPERO (CRD42024546509), PubMed, CINAHL, and Web of Science were searched for articles matching the study objective (pharmacists, motivational interviewing, and patient outcomes) from January 2014 to April 2024. Two reviewers independently screened titles, abstracts, and full texts. Data from included articles were extracted, synthesized categorically, and underwent quality assessment using the Mixed Methods Assessment Test. Results: Out of 356 identified articles, 45 were included. Randomized controlled trial designs implemented in ambulatory care or community pharmacies in the Global North were most common. Studies most frequently addressed diabetes, hypertension, and smoking cessation. Overall, MI interventions were associated with positive or neutral outcomes, with no studies reporting worsened results. Conditions such as hypertension and lipidemia and the need for nicotine cessation had positive outcomes, while diabetes and cardiovascular conditions contained both positive and neutral outcomes. Conclusions: Given its low implementation burden, MI represents a potential strategy for pharmacists and student pharmacists to improve patient outcomes, though future research should evaluate intervention fidelity and expand settings studied.

## 1. Introduction

Patients face a variety of health challenges associated with chronic diseases, including significant morbidity and mortality [1]. To prevent and lessen chronic disease burden, patients must make self-care-related behavioral changes, such as modifying their diet, integrating exercise into their routines, and taking medications. These changes can be challenging for patients to implement, often resulting in poor adherence to self-care practices [2,3]. One approach to aiding patients in making these changes is motivational interviewing (MI). MI is a patient-centered communication strategy aimed at improving health outcomes by facilitating behavior change [4]. While originally utilized within addiction care, it is now used across the patient care spectrum. In MI, patient decision-making is at the center of the behavior change process; providers must understand behavior change principles. They serve as guides to patients by walking them through thought processes that lead to behavior change, providing advice in relation to aspects that drive a specific patient’s motivation [5]. MI has been successfully utilized across a range of conditions, including vaccine hesitancy [6,7,8,9,10], cardiovascular conditions [11], and diabetes [12].

MI can be delivered as a stand-alone intervention or embedded as part of a larger bundled intervention or service, such as pharmacist-led services (i.e., comprehensive medication management, medication therapy management, and medication adherence). Given pharmacists’ roles in a variety of patient-facing settings, including community pharmacies and ambulatory care, they are well-positioned to address patient self-care adherence and utilize MI skills [13]. Many pharmacy curricula integrate MI as an essential patient communication skill [14,15], and multiple studies have provided evidence of the positive impact of student pharmacist and pharmacist-based MI patient interventions [8,16,17].

There are limited summaries regarding the outcomes of MI-based interventions by pharmacists and student pharmacists. While prior systematic reviews have examined pharmacist-led MI in specific disease contexts (e.g., cardiovascular disease) or for medication adherence alone [18,19], no review has broadly synthesized outcomes across conditions and the full spectrum of pharmacy-based providers, including student pharmacists. A systematic review is well-suited to synthesize this body of evidence, inform future research, and strengthen pharmacist-delivered patient care. Thus, the objective of this project is to describe the outcomes of MI interventions by pharmacists and student pharmacists.

## 2. Materials and Methods

To address the objective, a systematic review of the literature was conducted using PRISMA methodology [20] (see Table S1 for the PRISMA checklist). The search terms and inclusion criteria were established before beginning the process. The strategy was registered in PROSPERO (CRD42024546509). A research librarian was used to refine the search terms and process. Full inclusion and exclusion criteria can be found in Table 1, and an example search strategy can be found in Table S2. The inclusion criteria consisted of research studies (no review articles or commentaries), full-text availability, and being written in English. Meta-analyses, systematic reviews, and scoping reviews were excluded after searching the reference lists for any missing studies from the search. MI was included whether it was a standalone measure or embedded as part of a bundle of services. The search terms were determined to be (1) “motivational interviewing” or “directive counseling” or “counseling” and (2) “pharmacy student(s)” or “pharmacy” or “pharmacist.” These search terms were refined by a research librarian, who then tested the search strategy in different databases before finalizing. The databases utilized for these searches included PubMed, CINAHL, and Web of Science, and searches were conducted from 1 January 2014 to 9 April 2024. A 10-year period was chosen to provide a more contemporary overview of interventions, along with the rising increase in MI use among pharmacists during that time period. All references were then imported into Zotero for cleaning and uploaded into Covidence, a systematic review management system, for review.

Once the search was uploaded into Covidence and duplicates were removed, the screening process was started. Multiple articles by the same research group were included, as long as they presented different findings or a sub-analysis of the prior findings. The first step included two members of the group reviewing titles and abstracts of articles to see if they matched the inclusion criteria, with “yes” or “maybe” to move to the next stage and a “no” to exclude. A third member of the group (AC) resolved any disagreements about articles that should be included or excluded. The next step was the full-text review. In the full-text review, two members of the group read each article to ensure inclusion criteria were met. If yes, the articles moved to data extraction. If no, the reviewers selected “no” and a pre-specified reason for exclusion aligned with the exclusion criteria. A third member of the group (AC) managed any disagreements about the articles in this step as well. In the final step of the review process, two members of the research team performed data extraction from the full-text articles using a pre-specified template. The extracted data included: author, year of publication, country of publication, objective, study design, patient outcomes, and patient characteristics. Quality assessment was also performed at this step using the Mixed Methods Assessment Tool (MMAT) [21]. The MMAT was chosen because of its use across different study designs, which fit the broader scope of this systematic review. It has been previously used in systematic reviews with varying study designs [22,23], with the following scoring system [23]. Scores on the MMAT could range from 0 to 7, with higher scores indicating higher quality. This data was then examined by another member of the research team (AC) to ensure the accuracy of the completed work and resolve any discrepancies. Once completed, the results were synthesized into themes for reporting. The studies were then re-reviewed for categorization according to themes and verified by the entire research group. Then, a narrative synthesis was performed, as interventions, comparators, and outcome assessments were substantially different between studies.

## 3. Results

**Figure 1:**
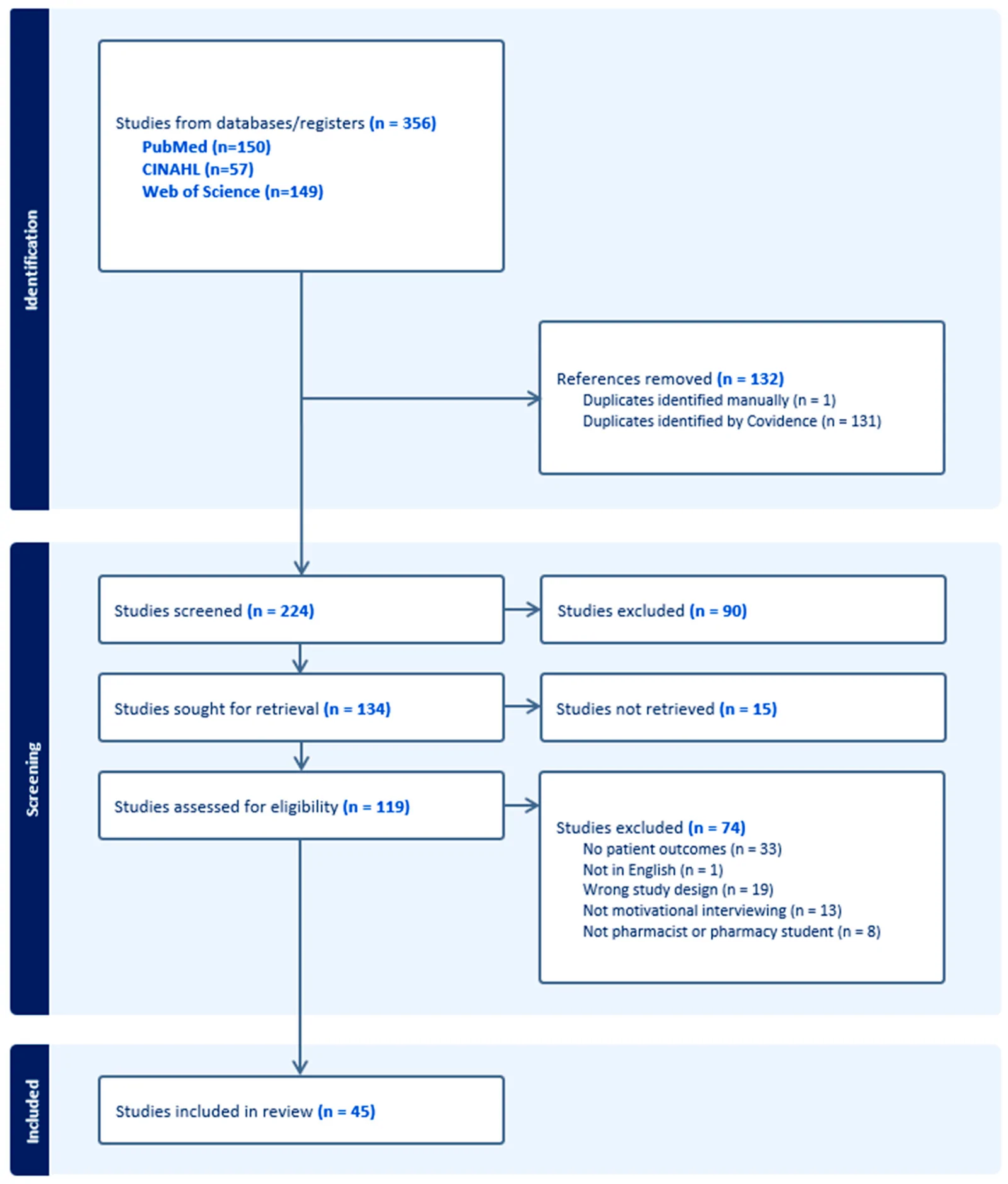
PRISMA flow diagram of included and excluded studies.

A total of 356 articles were initially identified for review, and after duplicates were removed, 224 articles underwent title and abstract screening. This produced 134 articles that received full-text review. Articles were excluded during this step for reasons such as lack of patient outcomes, lack of an available full-text article, wrong study design, not pharmacist-centered, and not MI (example excluded article [24]). A final total of 45 studies were included for data extraction. The breakdown of article screening and inclusion is shown in the PRISMA diagram (see Figure 1).

The average score for quality assessment of the articles used was 6.3, with 43/45 studies (96%) having a quality rating of 5 or higher (see Table 2).

All of the studies are summarized in detail in Table 4. Most of these studies were randomized controlled trials (*n* = 18, 40%) or quasi-experimental studies (*n* = 17, 37.8%). They were often conducted in the Global North, the United States (*n* = 22, 48.9%) or Europe (*n* = 15, 33.3%), after the year 2015 (*n* = 36, 80%). Studies evaluated pharmacist or pharmacy student MI interventions in settings such as ambulatory care clinics (*n* = 20, 44.4%), community pharmacies (*n* = 12, 26.7%), hospitals (*n* = 7, 15.6%), or managed care organizations (*n* = 6, 13.3%) (see Table 3). These studies included a variety of other interventions, such as group-based education classes [25], one-on-one patient appointments [26], medication list reviews [27], and interprofessional care [28,29,30].

An overview of the studies can be found in Table 4. Studies most commonly focused on utilizing MI for health conditions such as diabetes (*n* = 11), hypertension (*n* = 10), smoking cessation (*n* = 5), hyperlipidemia (*n* = 4), other cardiovascular conditions (*n* = 5), and kidney disease (*n* = 4). Most interventions involved multiple sessions rather than individual encounters and were delivered in person, though virtual and hybrid delivery methods were utilized. Most studies evaluated clinical outcomes of the MI interventions, followed by medication adherence and humanistic outcomes. Overall, studies reported a positive impact of the MI interventions, with no studies reporting a negative or worsened outcome. For instance, all studies evaluating the impact of MI on hypertension, smoking cessation, and hyperlipidemia outcomes had primarily positive outcomes reported. Studies that had neutral outcomes were focused on diabetes (*n* = 2), kidney disease (*n* = 1), other cardiovascular conditions (*n* = 2), or other clinical conditions (*n* = 6). Ten studies incorporated pharmacy students into the MI intervention [16,30,31,32,33,34,35,36,37,38]. These were distributed across managed care organizations (*n* = 5) [16,31,34,35,37], ambulatory care (*n* = 4) [30,32,33,36], and hospital (*n* = 1) [38] settings. Notably, the five studies conducted in the managed care organizations all evaluated a medication adherence outcome, and each reported positive results.

The specific measures assessed within each outcome category are summarized in Table 5. Measured outcomes were predominantly surrogate or process endpoints. Among the clinical outcomes, blood pressure was most common (*n* = 11), followed by A1c (*n* = 5), healthcare utilization (*n* = 5), and lipids (*n* = 4). Adherence was assessed mainly by adherence scales (*n* = 11) and proportion of days covered (*n* = 8), and humanistic outcomes focused on patient satisfaction (*n* = 7) and quality of life (*n* = 4).

### 3.1. Medication Adherence Outcomes

Most studies on medication adherence had positive outcomes (17/22, 77.3%). Studies utilized a variety of methods to assess medication adherence, including both self-report and more objective measures, so the definition of improvement in medication adherence may not be consistent across these results. For example, a brief, in-person MI intervention for diabetes found significant improvement in a direct measure of adherence, but no significant improvement in the self-perceived medication adherence [40]. Similarly, claims-based adherence measures, such as PDC and MPR, were positively impacted post-intervention [16,31,34,35,36,37,41]. These findings were not always consistent, as there were no differences post-intervention in stroke/TIA patients’ MPR [27].

Studies that used self-report measures of adherence often reported no or mixed changes in adherence, such as a rheumatoid arthritis intervention [25], an atrial fibrillation intervention [62], and a hypertension intervention [46]. A diabetes intervention had mixed findings with overall no differences but some potential benefits for patients who had more intervention sessions [47].

### 3.2. Clinical Outcomes

There were 23 of 32 studies (71.9%) evaluating clinical outcomes that had positive results. Studies looking at blood pressure lowering often had positive results [28,29,45,46], including single-encounter models [45], multi-encounter models [46], and team-based models [28,29]. Of note, a kidney-focused intervention with hypertension as a second outcome found no differences in blood pressure [39]. Smoking cessation interventions also had primarily positive outcomes, including higher quit rates in large RCTs [17] and smaller ambulatory care and hospital-based interventions [30,58,59]. Vaccine-focused interventions were mixed, with positive impacts seen in Hepatitis B [51] and overall vaccination rates [53], while others reported increased readiness to be vaccinated but no change in actual rates [52].

Studies evaluating glycemic changes had mixed results (positive [29,66], neutral [47]), which was similar to those evaluating healthcare utilization (positive [48,50], neutral [38,49]). Two ambulatory care-based studies with regular patient follow-up found significant improvements in A1c [28,29], but a larger phone-delivered intervention did not find significant improvements [47]. With healthcare utilization, an MTM intervention reduced ED visits and a discharge-based intervention reduced 30- and 180-day readmission rates. [50]. A pediatric asthma intervention, however, did not result in reduced healthcare visits [49], and an inpatient psychiatric intervention reduced ED visits only among those who received at least two sessions [38].

### 3.3. Humanistic Outcomes

Studies that evaluated humanistic outcomes had largely positive results (12/17, 70.6%). Often, these humanistic outcomes, such as patient satisfaction, acceptability, and confidence, were secondary outcomes, relying on self-reporting. Satisfaction with MI-based pharmacist encounters was consistently high in the studies, regardless of disease or delivery mode, including stroke/TIA [27], hemodialysis [64], and opioid misuse [61], indicating high acceptability for pharmacist-delivered MI interventions regardless of outcomes.

### 3.4. Variability Among Studies

Delivery mode was examined across the studies. Telephone-only MI interventions were effective in improving objective adherence measures, whether delivered by pharmacists or student pharmacists [16,31,34,35,36,37]. These most often occurred in the managed care setting [16,31,35,37,67]. In-person or a hybrid delivery (initially in-person, with phone follow-ups) was positive in certain diseases, such as blood pressure interventions [28,29,45,46]. No study directly compared the delivery mode of MI and its impact on outcomes.

There were also differences in findings between some of the study designs. For example, designs that did not include a usual care comparator (i.e., non-RCT) more frequently reported positive outcomes [28,29,44,48,56,57], while RCTS often found mixed or no change in outcomes [25,46,47,49,62]. It is unknown, however, the extent to which behavior-related communication styles are present within standard/usual care practices.

Finally, as previously noted, objective measures of adherence (claims data, refill data) were more likely to have positive findings associated with MI, while subjective measures were not.

## 4. Discussion

Pharmacists and student pharmacists implemented MI across 45 studies, at least 8 disease states, 4 practice settings, and multiple study designs. In this systematic review, the outcomes of the 45 MI interventions were mostly positive. These results emphasize the importance of implementing MI by pharmacists and pharmacy students in hospitals, clinics, and community pharmacies to benefit the health and medication adherence of their patients. The high methodological quality of most studies, i.e., higher MMAT scores based on clear research questions and appropriate reporting of all study elements, provides further support for these outcomes. Our findings are consistent with previous systematic reviews that reinforce the idea that pharmacist-led interventions are effective in improving outcomes and adherence, particularly in chronic disease states [18,19]. Our particular study is unique due to it being solely focused on MI.

The most common settings utilized across studies were ambulatory care (44%), with community pharmacies less common (27%). Pharmacy models in ambulatory care can lend to MI-based encounters with more time, established follow-up structures, and pharmacists embedded in an appointment-based disease management model. Alternatively, community pharmacies often have time constraints and “walk-in” patients. Examples across these studies can help pharmacists determine a feasible model, as positive impacts were seen in both models [8,10,53]. It is important to note that many of the studies had a multiple-session model, noting that the nature of MI does lend itself to relationship-building and follow-up rather than a single encounter for more complex disease states. The expansion of pharmacists in managed care organizations, where they may work to drive patient adherence to therapy, provides another avenue for a multi-session model. However, vaccine-based interventions can work in a single-encounter model [8,9,53] more readily than chronic disease interventions. Of note, delivery mode did matter, depending on setting. Telephone-only interventions were effective in managed care settings [16,31,34,35,37], while in-person and hybrid deliveries were more effective in other settings [28,29,45,68,69,70]. Direct comparison of delivery modes should be an area of future exploration to determine the most effective means of delivering MI in pharmacy settings.

Several patterns across the different studies may explain the heterogeneity of findings. These patterns should be further explored, as it will be important to assess these in order to determine the true impact of MI across disease states. First, how adherence was measured (objective or subjective) seemed to have resulted in different outcomes, even when examined among the same patients in the same study [40]. Self-report measures can be subjective due to recall bias and social desirability bias; thus, they may not adequately capture the behavioral change. Secondly, health conditions that require multiple behavioral elements and often have multiple comorbidities may be more challenging to measure, determine the impact of an intervention, and change with brief or limited MI-based interventions. For example, diabetes and cardiovascular conditions more frequently had a neutral outcome, as compared to hypertension, smoking cessation, and hyperlipidemia, which had no neutral outcomes. Perhaps the complexity of these conditions and the corresponding self-care measures needed [71] or other barriers, such as the social determinants of health [72], contributed to this outcome. It may take more than MI to elicit behavior change when diseases are complex rather than more behavior-oriented conditions like smoking cessation. This would be consistent with the findings of Werremeyer 2019, which found that at least two sessions were needed for a change to occur [38]. Further, this could mean that there is a dose–response relationship between MI interventions and behavioral change. This is an area of further exploration within pharmacy-based MI interventions.

Study design also may have contributed to the differences seen among studies. Single-arm studies, cohort studies, and non-RCT experimental designs often found improvements [28,29,44,48,56,57], while RCTS with a comparator group (usual care) did not or found mixed results [25,46,47,49,62]. The RCTs were often in an ambulatory care setting, with the comparator being provider-based usual care. Thus, it may be that MI does not have as much of an impact on patient outcomes as simply providing usual care, or providers could be providing some elements of behavior-based interventions as part of usual care practices. This should be further explored in studies to determine not only whether MI works but whether usual care has also evolved to include more behavioral interventions, particularly when the comparator is ambulatory care practice.

Notably, none of the 45 studies reported a negative or worsened outcome. Thus, the risk versus benefit of implementing MI could be fairly high, resulting in equivalent outcomes at worst. Indeed, most studies found positive outcomes. Given the ability to implement MI without a formulary change, collaborative practice agreement, or prescribing authority, the barrier to implementation is lower than some other practice changes. For example, interventions can be brief, limiting the time investment [54]. While sites will need to invest in training their staff, several studies outlined their training models [17,55], which could provide some guidance. Sites should also identify how best to implement this in their workflow; successful integration in large chain pharmacy settings could provide an example model [53]. If sites have limited resources, student pharmacists may aid in lessening the investment needed. Several studies included in this review examined the role of pharmacy students leading interventions and found them to be effective [16,30,36]. Pharmacy programs often include MI as part of their curriculum [14], providing a trained workforce to provide MI interventions.

### Limitations

There are several limitations to this systematic review. First, few studies described measures taken to assess the fidelity of the MI interventions (i.e., how well the pharmacists implemented the MI intervention as described) [73]. Additionally, while these studies included an MI component, it is important to note that other interventions often happened alongside them. It is therefore difficult to extrapolate how much of the impact seen in a study is due to the MI component versus another factor [41]. Secondly, while we found a lack of negative outcomes across studies, this could be due to publication bias. Researchers may not have published findings that did not show a benefit. Thirdly, studies were heterogeneous in nature, limiting the ability to truly compare outcomes. Multiple studies by the same author group were included, which may present different components of the results from the same study. This could have introduced bias into the findings. Finally, studies were mainly implemented in the Global North (the United States and Europe), limiting generalizability to other pharmacy settings in the Global South. Future work should evaluate the impact of standardized, high-fidelity MI interventions, the implementation of MI and its outcomes in underrepresented settings (such as hospital systems and managed care organizations), other disease states that require behavior change, and the number of sessions needed for change to occur (single MI intervention versus multiple sessions).

## 5. Conclusions

Our systematic review of pharmacist-delivered MI interventions was consistently associated with positive or neutral outcomes, notably in hypertension, smoking cessation, and hyperlipidemia. No studies reported negative outcomes. Given the low implementation barrier for MI-based interventions, MI could be a scalable, low-resource strategy for pharmacists to positively impact the health of communities and improve patient outcomes. Future research should explore the fidelity of MI-based interventions as well as expand efforts across a variety of diseases and settings.

## Figures and Tables

**Table 1:** Inclusion and exclusion criteria.

Criteria	Inclusion	Exclusion
Year of Publication	1 January 2014–9 April 2024	Else
Type of Publication	Research Studies ^1^	Other types of publications (Review article, commentary) ^2^
Access	Full-text	Not available in full text
Language	English	All other languages
Content	Population: Pharmacist/Student Pharmacist; Intervention: Motivational Interviewing; Outcome: clinical, economic, or humanistic	Else

^1^ Examples include: RCT, Cohort Study, Cross-Sectional Study, Chart Review, Interventional Study. ^2^ Meta-analyses, systematic reviews, and scoping reviews were excluded after reviewing the reference lists for any missing articles.

**Table 2:** Quality assessment of extracted studies using the Mixed Methods Appraisal Tool (MMAT).

Author (Year)	Clear Research Question	Data Address Research Question	MMAT Summary Evaluation of Quality
Abughosh (2017)	Yes	Yes	7
Abughosh (2019)	Yes	Yes	6
Bandiera (2022)	Yes	Yes	6
Bandiera (2024)	No	Yes	7
Berenbrok (2023)	Yes	Yes	6
Brackett (2015)	Yes	Yes	6
Buckley (2017)	Yes	Yes	7
Cantillana-Suarez (2021)	Yes	Yes	6
Caponnetto (2017)	Yes	Yes	7
Chen (2022)	Yes	Yes	6
Cochran (2019)	Yes	Yes	7
Coley (2020)	No	Yes	4
Deeks (2019)	Yes	No	2
DePatis (2019)	Yes	Yes	7
Dhital (2015)	Yes	Yes	7
Dobrinas (2014)	Yes	Yes	6
Ekong (2020)	Yes	Yes	5
Goldberg (2019)	Yes	Yes	6
Hedegaard (2014)	Yes	Yes	7
Hedegaard (2015)	Yes	Yes	7
Hedegaard (2016)	Yes	Yes	6
Hurst (2021)	Yes	Yes	7
Ikolaba (2023)	Yes	Yes	5
Ismail (2019)	Yes	Yes	6
Jaffray (2014)	Yes	Yes	5
Jalal (2016)	Yes	Yes	7
Kim (2020)	Yes	Yes	6
Klamerus (2014)	Yes	Yes	7
Lauffenburger (2019)	Yes	Yes	6
Majd (2024)	Yes	Yes	6
Mohan (2023)	Yes	Yes	7
Moreno (2021)	Yes	Yes	5
Morillo-Verdugo (2019)	Yes	Yes	6
Mosquera (2021)	Yes	Yes	7
Okada (2018)	Yes	Yes	7
Okuyan (2021)	Yes	Yes	7
Osbaugh (2022)	Yes	Yes	7
Ostbring (2021)	Yes	Yes	7
Paneerselvam (2023)	Yes	Yes	7
Paranjpe (2020)	Yes	Yes	6
Ravn-Nielsen (2018)	Yes	Yes	7
Shiga (2022)	Yes	Yes	7
Stewart (2014)	Yes	Yes	7
Werremeyer (2019)	Yes	Yes	6
Zwikker (2014)	Yes	Yes	6

**Table 3:** Characteristics of included articles.

Item	N (%) (*n* = 45)
Year of Study Publication	
2015 or before	9 (20%)
After 2015	36 (80%)
Country	
United States	22 (48.9%)
Australia	2 (4.4%)
Europe	15 (33.3%)
Asia	3 (6.7%)
Middle East	2 (4.4%)
Africa	1 (2.2%)
Study Design	
Randomized Controlled Trial	18 (40%)
Non-randomized Controlled Trial	2 (4.4%)
Quasi-experimental	17 (37.8%)
Cohort	5 (11.1%)
Qualitative	1 (2.2%)
Mixed Methods	2 (4.4%)
Pharmacy Setting	
Ambulatory Care	20 (44.4%)
Community	12 (26.7%)
Hospital	7 (15.6%)
Managed Care Organizations	6 (13.3%)
Number of Participants (median, range)	
Total participants, all studies	20,006 (140, 19–5209)
Single-arm studies (*n* = 20)	
Total N	3377 (70, 19–1400)
Studies with a control group (*n* = 25)	
Total N	16,629 (366, 30–5209)
Intervention arm n	6062 (152, 15–1569)
Participants receiving intervention, all studies	9439 (104, 15–1569)

**Table 4:** Summary of included studies.

Author Year Country Pharmacy Setting	Study Design Control Group—Yes/No Pharmacy Personnel N (of Participants)	Disease State/Condition	MI Intervention Delivery Mode	Primary Outcome Measured	Key Results
Abughosh 2017 United States Managed Care Organization	Quasi-Experimental Control Group—Yes Student Pharmacist N = 743 (Control = 495, Intervention = 248)	Hypertension Diabetes	Initial call + monthly follow-up calls for 6 months to identify barriers and achieve therapy goals Virtual (phone)	PDC Medication discontinuation	Patients completing the initial call and at least 2 follow-ups were less likely to discontinue (OR = 0.29; 95% CI = 0.15–0.54; *p* < 0.001) and more likely to be adherent in the linear regression model (β = 0.0604, *p* < 0.001) and the logistic regression model (OR = 1.53; 95% CI = 1.02–2.28; *p* = 0.009). Other factors significantly associated with better adherence included higher baseline PDC and number of medications.
Abughosh 2019 United States Managed Care Organization	Quasi-Experimental Control Group—Yes Student Pharmacist N = 456 (Control = 304, Intervention = 152)	Hyperlipidemia	Initial call + 2 follow-up calls (monthly) to identify barriers and enhance adherence Virtual (phone)	PDC Medication discontinuation	Mean PDC for the intervention group (0.67 ± 0.3) was significantly higher than the control (0.55 ± 0.4; *p* < 0.001). The intervention group was also less likely to discontinue (OR = 0.38; 95% CI = 0.19–0.76) and more likely to be adherent in the linear regression model (β = 12.4; *p* < 0.001) as well as in the logistic regression model (OR = 1.87; 95% CI = 1.18–2.95). Previous adherence trajectories were significantly associated with adherence in the follow-up.
Bandiera 2022 Europe Ambulatory Care	Mixed Methods (qualitative portion reported here) Control Group—Yes Pharmacist N = 275 (Control = 202, Intervention = 73), but *n* = 14 for interview	Kidney Disease (comorbidity medication adherence)	15–20 min MI intervention at each appointment In-person pre-COVID Virtual (phone) post-COVID	Satisfaction	The included patients who were interviewed (*n* = 14) found the interprofessional intervention useful to improve their medication management, support medication literacy, and motivation.
Bandiera 2024 Europe Ambulatory Care	RCT Control Group—Yes Pharmacist N = 275 (Control = 202, Intervention = 73)	Kidney Disease (comorbidity medication adherence)	15–20 min MI intervention at each appointment for 6 months (Group B) or 12 months (Group A) In-person pre-COVID Virtual (phone) post-COVID	Adherence BP	Adherence increased during intervention and decreased during follow-up No difference in blood pressure. No difference in statin adherence. 12-month adherence: Antidiabetic medication higher in group A versus group B (93.8% versus 86.8%; 95% CI: 5.7%; 8.3%). Antihypertensive medication higher in group A versus B (97.9% versus 92.1%; 95% CI: 4.8%; 6.7%). 24-month adherence: Antidiabetic and antihypertensive drugs: higher in group A versus B (88.6% versus 85.6%; 95% CI: 1.7%; 4.4%; 94.4% versus 85.9%; 95% CI: 6.6%; 10.7%, respectively).
Berenbrok (2023) United States Community Pharmacy	Non-RCT Control Group—Yes Pharmacist N = 5209 (Control = 3640, Intervention = 1569)	Diabetes (HepB vaccination)	MI intervention at medication pick-up In-person	Rate of initiation of Hepatitis B vaccine series	Statistically significant increase in HepB vaccination (3.7%) in intervention vs. control. 40 of 65 patients (61.5%) completed the vaccination series.
Brackett (2015) United States Community Pharmacy	Quasi Experimental Control Group—No Pharmacist N = 19	Vaccines	MI intervention at medication pick-up In-person	Vaccine acceptance Patient readiness to receive immunizations	No change in immunization rates. Readiness improved: hepatitis B (*p* = 0.001) and pneumococcal (*p* = 0.033) vaccines.
Buckley 2017 United States Ambulatory Care	Cohort Control Group—No Student Pharmacist N = 139	Smoking Cessation	Multidisciplinary MI intervention, with student pharmacists addressing NRT Pharmacy-only sites: student pharmacists provided all elements In-person with 5 phone follow-ups over 10 days	Ability to follow up Interest in quitting Quit rates	Intervention delivered in a homeless population 19 (13.7%) successfully contacted the smoking cessation quitline. 2 quit at follow-up phone calls. Patients reported high baseline confidence, knowledge, and willingness related to quit attempts.
Cantillana-Suárez 2021 Europe Hospital Pharmacy	Quasi-Experimental Control Group—No Pharmacist N = 349	HIV	MI follow-up by pharmacists to address adherence In-person	Acceptance of pharmacist recommendations Medication adherence	High recommendation acceptance by doctors and patients [336 (97.7%) and 321 (93.3%)]. Improved adherence rate to antiretroviral therapy post-intervention (85.6% ± 33.7% vs. 96.4% ± 17.7%; *p* < 0.001).
Caponnetto 2017 Europe Community Pharmacy	RCT Control Group—Yes Pharmacist N = 187 (Control = 63, Intervention = 124)	Smoking Cessation	MI intervention by pharmacists for patients who sought advice on smoking cessation or bought NRT In-person	Quit rate	At week 24, higher quit rate in intervention vs. control (12.2% vs. 1.6%).
Chen 2022 United States Ambulatory Care	Quasi Experimental Control Group—No Pharmacist, Student Pharmacist N = 19 patients, 11 providers	Pain Management	Provider (including pharmacists) set pain management goals using MI; biweekly MI follow-up with student pharmacists to evaluate pain management and goal achievement In-person with phone follow-ups	Provider knowledge/confidence Number of opioid prescriptions MME	Increased provider confidence but not satisfaction. Patients (*n* = 19) were able to set and accomplish 20 goals throughout the phone call intervention. One-year pre-intervention vs. intervention period: Opioid prescriptions significantly decreased from 569 to 368. MME decreased from 26.8 to 26.4.
Cochran 2019 United States Community Pharmacy	RCT Control Group—Yes Pharmacist N = 32 (Control = 17, Intervention = 15)	Pain Management	MI intervention during 30–45 min session, followed up by a patient navigator In-person with phone follow-ups	Completion rates Patient satisfaction Prescription Opioid Misuse Index Pain levels	High completion rates. Intervention satisfaction: ≥4.2 (max = 5) level of satisfaction with the pharmacist-led session. 92.4% were satisfied with navigation sessions. Intervention outcomes: Greater improvements in misuse (ITT: Adjusted Odds Ratio [AOR] = 0.13; 95% CI = 0.05, 0.35, *p* < 0.001. NUMSESS: AOR = 0.05; 95% CI = 0.01, 0.25; *p* < 0.001). Greater improvements in pain (ITT: B = 8.8, 95% CI = −0.95, 18.5, *p* = 0.08; NUMSESS: B = 14.0, 95% CI = 3.28, 24.8, *p* = 0.01).
Coley 2020 United States Community Pharmacy	Quasi-Experimental Control Group—No Pharmacist N = 99 pharmacies	Vaccines	MI intervention monthly based on eligible vaccines In-person with phone follow-ups	Vaccination rate	33% increase in vaccinations vs. prior year, with 3 out of 4 vaccines improving: 45% for influenza. 31% for pertussis. 7% for pneumococcal vaccinations. Decrease of 5% for herpes zoster vaccinations.
Deeks 2019 Australia Ambulatory Care	Quasi-Experimental Control Group—No Pharmacist N = 66	Smoking Cessation	MI-based quit coaching and follow-up as needed In-person	Quit rate at 6 months	Quit rate at 6 months: 30% (20/66). Biochemically verified smoking abstinence rate: 20% (13/66). Successful quit attempts were associated with: Varenicline recommendation (69% vs. 25%). Increased median number of practice pharmacist consultations (4 vs. 2 per patient).
DePatis 2019 United States Ambulatory Care	RCT Control Group—Yes Pharmacist, Student Pharmacist N = 30 (Control = 15, Intervention = 15)	Diabetes	One or two MI sessions with pharmacist during clinic visit In-person	Urinary albumin screening ACE/ARB initiation	No significant differences.
Dhital 2015 Europe Community	RCT Control Group—Yes Pharmacist N = 407 (Control = 202, Intervention = 205)	Alcohol Use	MI session with pharmacist to discuss AUDIT scores and the role of drinking In-person	AUDIT scores	Reduced AUDIT score but not significant (intervention minus control −0.57).
Dobrinas 2014 Europe Hospital	Quasi-Experimental Control Group—No Pharmacist N = 40	Smoking Cessation	MI intervention during 30–45 min session, second visit depending on LOS In-person	Readiness to quit Smoking status	One-month post-discharge: 53% improved readiness to quit. 33% abstinent.
Ekong 2020 United States Ambulatory Care	Quasi-Experimental Control Group—No Pharmacist, Resident N = 36	Diabetes	3 brief MI interventions in 3 months In-person	Medication adherence Clinical outcomes (BP, A1c)	Significant improvement in measured adherence (*p* = 0.010). SDSCA-MS score improved but was not significant. Improved DBP (*p* = 0.034). No change in SBP or A1c.
Goldberg 2019 United States Hospital	Qualitative Control Group—No Pharmacist N = 20	Fall risk	Bedside brief MI intervention on medications and fall risk In-person	Perceptions of MI	Positive perceptions of MI.
Hedegaard 2014 Europe Hospital	RCT Control Group—Yes Pharmacist N = 211 (Control = 107, Intervention = 104)	Stroke/TIA	30 min MI-based intervention with follow-ups at 1 week, 2 months, and 6 months In-person with phone follow-ups	MPR Patient satisfaction, knowledge, confidence	From 3 to 12 months, the MPR decreased by 5% (*p* < 0.05) in intervention and 9% (*p* < 0.05) in control, no significant difference. Drug-related problems identified in 1/3 of patients. High satisfaction with the intervention, ~50% increased knowledge, ~1/3 increased confidence.
Hedegaard 2015 Europe Ambulatory Care	RCT Control Group—Yes Pharmacist N = 532 (Control = 292, Intervention = 240	Hypertension	Initial MI-based intervention with follow-ups at 1 month and 6 months In-person with phone follow-ups	MPR	Lower nonadherence at 12 months in intervention (MPR < 0.80; 20.3%), vs. control (30.2%) (risk difference −9.8; 95% confidence interval [CI], −17.3, −2.4). Median MPR (interquartile range): intervention = 0.93 (0.82–0.99) vs. control = 0.91 (0.76–0.98). *p*= 0.02.
Hedegaard 2016 Europe Ambulatory Care	RCT Control Group—Yes Pharmacist N = 156	Hypertension	Initial MI-based intervention with follow-ups at 1 month and 6 months In-person with phone follow-ups	Patient satisfaction	~50% of patients reported increased focus on lifestyle change. 21–39% reported increased knowledge, confidence and skills related to medications and improved QOL.
Hurst 2021 United States Ambulatory Care	Quasi-Experimental Control Group—No Pharmacist N = 95	Hypertension Smoking Cessation Diabetes Hyperlipidemia COPD Heart Failure	30 min MI intervention with pharmacist and then a health coach, then seen monthly by both In-person	A1c BP Lipids	At 1 year, significant improvements in: A1c (mean ± SD, 8.55 ± 2.58 to 7.04 ± 1.12, *p* < 0.001). SBP (136.79 ± 20.04 to 123.15 ± 16.81, *p* < 0.001). DBP (87.94 ± 12.28 to 78.64 ± 10.98, *p* < 0.001). Total cholesterol (198.25 ± 52.47 to 183.55 ± 47.22, *p* = 0.014). LDL cholesterol (115.74 ± 43.56 to 105.92 ± 39.27, *p* = 0.040).
Ikolaba 2023 Africa Community Pharmacy	Mixed Methods Control Group—No Pharmacist N = 104	Diabetes	6-month MI intervention with pharmacist to develop goals Virtual	BMI, waist circumference, and fasting plasma glucose Adherence Patient Satisfaction	All clinical and adherence outcome measures showed statistically significant improvements (*p* < 0.05). Patients reported satisfaction with intervention.
Ismail 2019 Middle East Ambulatory Care	Quasi-Experimental Control Group—No Pharmacist N = 67	Kidney Disease	Medication adherence interview at 1, 2, 4, 6 months with CMR at 3, 5 months MI intervention beginning in month 3 In-person	Adherence SBP LDL MRP	No change in adherence. No change in SBP and LDL. Significant decrease in MRP: Months 3–5 (*p* = 0.002), change from 44.9% (95 confidence interval [CI]: 40.4–49.3) to 29.8% (95 CI: 25.6–34.3) at Month 5. Drug use without indication was the most frequent MRP (23.9%).
Jaffray 2014 Europe Community Pharmacy	RCT Control Group—Yes Pharmacist N = 542 (Control = 247, Intervention = 295)	Alcohol, Drug Use	MI intervention at multiple visits In-person	Illicit heroin use	No significant difference between groups. Treatment retention higher in intervention group, not significant (88% cf. 81%; *p* = 0.34); no significant difference between groups in treatment satisfaction, although this improved significantly in intervention (*p* < 0.05). Intervention patients perceived pharmacist MI useful (*p* < 0.05).
Jalal 2016 Europe Community Pharmacy	Non-RCT Control Group—Yes Pharmacist N = 71 (Control = 39, Intervention = 32)	ACS	Follow-up: 15–20 min MI intervention at 2 weeks post-discharge In-person or phone	Medication adherence Medication beliefs	Significant difference in adherence between intervention and control: At 3 months: (M = 7.7, SD = 0.56) vs. (M = 7.0, SD = 1.85), *p* = 0.026. At 6 months (M = 7.5, SD = 1.47) vs. (M = 6.1, SD = 2.09), *p* = 0.004).
Kim 2020 United States Ambulatory Care	Quasi-Experimental Control Group—No Pharmacist N = 50	Hypertension Diabetes	Initial team-based MI visit with follow-up phone calls monthly until goals reached, then less frequently In-person, with follow-up phone calls	BP A1c	Significant reduction at 1 year in: SBP [151.5 mm Hg vs. 141.8 mm Hg, −9.7 mm Hg difference, 95% confidence interval (CI) −6.19 to −13.19, *p* < 0.001]. A1C (9.6% vs. 8.6%, −1.0% difference, 95% CI −0.49 to −1.39, *p* < 0.001). At each visit, mean SBP and A1C were significantly lower.
Klamerus 2014 United States Ambulatory Care	Cohort Control Group—No Pharmacist N = 458	Hypertension (diabetic patients)	MI encounters with pharmacist In-person	BP target attainment	Short-term success: ~90% of patients. Long-term success: 28% of patients.
Lauffenberger 2019 United States Managed Care Organization	RCT Control Group—Yes Pharmacist N = 1400 (Control = 700, Intervention = 700)	Diabetes	At least one MI intervention over the phone	A1c PDC	Change in HbA1c (from baseline): Control: 0.79 (SD:2.01). Intervention: −0.75 (SD:1.76). Difference: +0.04, 95% CI: −0.22, 0.30. As-treated analysis: intervention improved control (−0.48, 95% CI: −0.91, −0.05). No significant differences in adherence. Qualitative findings: indicated need to further address patient barriers.
Majd 2024 United States Managed Care Organization	Cohort Control Group—Yes Student Pharmacist N = 720 (Control = 480, Intervention = 240)	Hypertension Diabetes	Initial MI-based intervention with 5 follow-up phone calls Virtual	PDC	Intervention: less likely to have a slow decline in adherence (OR: 0.627 [0.401–0.981]).
Mohan 2023 United States Managed Care Organization	RCT Control Group—Yes Student Pharmacist N = 720 (Control = 480, Intervention = 240)	Hypertension Diabetes	Initial MI-based 15 min phone call with follow-up calls as needed Virtual	PDC	Intervention group had improved adherence vs. control: At 6 months (β = 0.06; *p* = 0.03). At 12 months (β = 0.06; *p* = 0.02 and OR: 1.46; 95% CI 1.05–2.04, respectively).
Moreno 2021 United States Ambulatory Care	Quasi-Experimental Control Group—Yes Pharmacist N = 2592 (Control = 1994, Intervention = 648)	Diabetes	Initial MI-based MTM consultation (up to 60 min), with follow-ups of 15–30 min In-person	ED/Hospitalization rates	ED hospitalization rate decreased from an adjusted mean monthly rate of 0.09 to 0.07 (*p* = 0.035), predicted reduction of 21% in ED visits. Predicted 3.2% reduction in hospitalizations over time, not significant.
Morillo-Verdugo 2019 Europe Ambulatory Care	Quasi-Experimental Control Group—No Pharmacist N = 140	HIV	Individualized interventions + MI and pharmacotherapeutic follow-up outside of consults In-person	Adequate adherence	Significant improvement in % of patients with adequate adherence, 18.4% (*p* = 0.035).
Mosquera 2021 United States Ambulatory Care	RCT Control Group—Yes Pharmacist N = 63 (Control = 29, Intervention = 34)	COPD Asthma	MI-based intervention by pharmacists for refills In-person and virtual	Healthcare visits	No significant improvement in reduced healthcare visits.
Okada 2018 Asia Community Pharmacy	RCT Control Group—Yes Pharmacist N = 125 (Control = 61, Intervention = 64)	Hypertension	MI-based intervention, with an initial visit and goal setting and achievement at subsequent visits (3 visits) In-person	SBP	Significant decrease in morning SBP: −6.0 mmHg vs. control (95% confidence interval [CI]: −11.0 to −0.9, *p* = 0.021). Mixed effects model: −4.5 mmHg (95% CI: −8.5 to −0.6, *p* = 0.024).
Okuyan 2021 Middle East Community Pharmacy	Quasi-Experimental Control Group—No Pharmacist N = 52	Older Adults	MI-based intervention to promote medication adherence, with follow-up calls to address MRPs In-person with follow-up phone calls	Medication adherence QOL	Significant improvements (*p* < 0.05) in: Medication adherence, 51.9% to 75%. QOL scores: 51.7 to 53.4.
Osbaugh 2022 United States Ambulatory Care	Quasi-Experimental Control Group—Yes Student Pharmacist N = 140 (Control = 82, Intervention = 58)	Hyperlipidemia	MI-based phone call intervention to address statin benefits Virtual	PDC	At 6 months, intervention group vs. control: Significant increase in mean PDC (12.8% vs. 0.7%, *p* = 0.004). 66% adherent vs. 41% [*p* = 0.005, adjusted OR = 2.46 (1.20–5.05)].
Östbring 2021 Europe Ambulatory Care	RCT Control Group—Yes Pharmacist N = 316 (Control = 157, Intervention = 159)	CAD	60 min MI-based encounter, 3 months after discharge In-person	LDL BP Adherence	No significant difference in target LDL levels. More intervention than control patients were adherent to cholesterol-lowering drugs (88 vs. 77%; *p* = 0 .033) and aspirin (97 vs. 91%; *p* = 0.036).
Paneerselvam 2023 Asia Hospital Pharmacy	Quasi-Experimental Control Group—No Pharmacist N = 63	Kidney Disease	15–20 min MI-based encounter at months 3, 6, 9 In-person with phone follow-ups	Medication adherence	Significant increase in medication adherence scores related to patient attitude and additional illness/pill overload (*p* < 0.05). MI increased intrinsic positive attitude by resolving ambivalence.
Paranjpe 2020 United States Managed Care Organization	Cohort Control Group—Yes Student Pharmacist N = 456 (Control = 304, Intervention = 152)	Hyperlipidemia	Initial call + 2 follow-up calls (monthly) to identify barriers and enhance adherence Virtual (phone)	Medication adherence	Furthermore, the predictors associated with the postintervention adherence trajectories included MoI intervention, prescriber specialty, presence of diabetes, presence of congestive heart failure, Centers for Medicare & Medicaid Services risk score, and preintervention adherence trajectories.
Ravn-Nielsen 2018 Europe Hospital Pharmacy	RCT Control Group—Yes Pharmacist N = 1499 (Control = 503, Intervention = 996)	Any	30 min MI session with follow-up at 1 week and 6 months post-discharge In-person with phone follow-ups	Readmission ED visits	Intervention significantly improved: 30-day readmissions (hazard ratio [HR], 0.62; 95% CI, 0.46–0.84). 180-day readmissions (HR, 0.75; 95% CI, 0.62–0.90). Nonsignificant reduction in: 30-day drug-related readmissions (HR, 0.65; 95% CI, 0.39–1.09). 180-day drug-related readmissions (HR, 0.80; 95% CI, 0.59–1.08). Deaths (HR, 0.83; 95% CI, 0.22–3.11). NNT = 12 (vs. usual care).
Shiga 2022 Asia Ambulatory Care	RCT Control Group—Yes Pharmacist N = 268 (Control = 134, Intervention = 134)	Atrial Fibrillation	MI-based education vs. usual medication counseling In-person	Medication adherence	No significant difference in medication use. Adherence to apixaban significantly improved in men in MI intervention (β = 0.219; *p* = 0.012).
Stewart 2014 Australia Community Pharmacy	RCT Control Group—Yes Pharmacist N = 366 (Control = 188, Intervention = 178)	Hypertension	Multiple MI-based sessions to enhance adherence In-person	Medication adherence	No significant difference in overall adherence. Intervention improved: SBP mean reduction (10.0 mmHg vs. 4.6 mmHg; *p* = 0.05). Proportion of patients who were non-adherent at baseline and adherent at 6 months: 22.6% (95% CI 5.1–40.0%) vs. control (61.8% vs. 39.2%, *p* = 0.007). For patients with baseline BP above target, systolic BP [by 7.2 mmHg (95% CI 1.6–12.8 mmHg); (*p* = 0.01)]. For participants non-adherent at baseline and above target BP, adherence at 6 months (56.8% vs. 35.9%, *p* = 0.039).
Werremeyer 2019 United States Hospital Pharmacy	Cohort Control Group—Yes Pharmacist, Student Pharmacist N = 583 (Control = 285, Intervention = 298)	Psychiatric Conditions	Twice-weekly, MI-based medication education In-person	Readmission ED visits	Intervention significantly reduced: ED visits for psychiatric reasons (*p* = 0.0433), if at least two sessions. No change for high-risk or other patients.
Zwikker 2014 Europe Ambulatory Care	RCT Control Group—Yes Pharmacist N = 366 (Control = 188, Intervention = 178)	Rheumatoid Arthritis	Two MI-based group sessions one week apart In-person	Medication beliefs Medication adherence	No differences between groups in most outcomes. At 12 months, intervention: had less strong necessity beliefs about medication than participants in the control arm (b: −1.0 (95% CI: −2.0, −0.1)).

RCT = randomized controlled trial, PDC = proportion of days covered, BP = blood pressure, MME = morphine milligram equivalent, AUDIT = Alcohol Use Disorders Identification Test, LOS = length of stay, SDSCA-MS: Summary of Diabetes Self-Care Activities-Medication Subscale, DBP = diastolic blood pressure, SBP = systolic blood pressure, TIA = transient ischemic attack, MPR = medication possession ratio, QOL = quality of life, COPD = Chronic Obstructive Pulmonary Disease, SD = standard deviation, LDL = low-density lipoprotein, ACS = Acute Coronary Syndrome, MRP = medication-related problem, MTM = medication therapy management, ED = emergency department, CAD = coronary artery disease.

**Table 5:** Specific outcome measures assessed across included studies.

Outcome Category	Specific Measure	N (*n* = 45)
Clinical	Blood pressure (SBP/DBP) [28,29,39,40,41,42,43,44,45,46]	11
	Glycemic: A1c [28,29,40,42,43,47]	5
	Healthcare utilization (ED visits, readmissions, hospital days) [38,41,48,49,50]	5
	Lipids (LDL, total cholesterol) [26,38,42,43]	4
	Mortality/cardiovascular composite endpoint [27,41,50]	3
	Vaccination: rate/uptake [51,52,53]	3
	Alcohol/drug use (AUDIT, heroin use) [54,55]	2
	BMI/waist circumference [45,56]	2
	Medication-related problems (count) [42,57]	2
	Smoking abstinence: eCO-verified [17,58]	2
	Smoking abstinence/readiness: self-reported [58,59]	2
	Glycemic: fasting plasma/blood glucose [56]	1
	HIV viral load/CD4 [60]	1
	Opioid prescribing (prescription count, MME) [32]	1
	Serum phosphate [42]	1
	Vaccination: series completion [51]	1
	Other clinical (single-study measures) [28,33,39,40,49,61,62]	7
Medication Adherence or Health Adherence	Adherence scales (Morisky/MMAS, GMAS, SDSCA, MARS, CQR, TABS) [25,26,40,43,45,46,56,57,60,63,64]	11
	PDC [16,31,34,35,36,37,47,60]	8
	MPR [25,27,41,65]	4
	Medication beliefs (BMQ)/necessity-concern [25,26,43,57]	4
	Electronic monitoring (EM/MEMS) [39,62]	2
Humanistic	Patient satisfaction [27,32,55,56,57,61,65]	7
	Quality of life (SF-12, EQ-VAS/EQ-5D, ADDQoL, OPQOL) [40,45,56,57]	4
	Confidence/knowledge [27,30,32]	3
	Patient activation (PAM) [56,63]	2

Key: systolic blood pressure (SBP), diastolic blood pressure (DBP), ED (Emergency Department), LDL (low-density lipoprotein), AUDIT (Alcohol Use Disorders Identification Test), BMI (body mass index), MME (morphine milligram equivalent), MMAS (Morisky Medication Adherence Scale), GMAS (General Medication Adherence Scale), SDSCA (Summary of Diabetes Self-Care Activities), MARS (Medication Adherence Report Scale), CQR (Compliance Questionnaire for Rheumatology), TABS (Treatment Adherence in Bipolar Spectrum), PDC (proportion of days covered), MPR (Medication Possession Ratio), BMQ (Beliefs about Medications Questionnaire), SF-12 (Short Form 12 Health Survey, EQ-VAS/EQ-5D (EuroQol Visual Analogue Scale/EuroQol 5-Dimension), ADDQoL (Audit of Diabetes-Dependent Quality of Life), OPQOL (Older People’s Quality of Life Questionnaire), PAM (Patient Activation Measure).

## Data Availability

No new data were created or analyzed in this study. Data sharing is not applicable to this article.

## Supplementary Materials

The following supporting information can be downloaded at: https://www.mdpi.com/article/10.3390/pharmacy14060136/s1, Table S1: PRISMA 2020 Main Checklist for the Study; Table S2: Search Strategy Example.
